# High dose chemotherapy and autologous bone marrow transplantation in refractory Hodgkin's disease.

**DOI:** 10.1038/bjc.1986.127

**Published:** 1986-06

**Authors:** T. Philip, J. Dumont, F. Teillet, D. Maraninchi, N. C. Gorin, M. Kuentz, J. L. Harousseau, M. Marty, R. Pinkerton, P. Herve

## Abstract

Seventeen patients with Hodgkin's disease (HD) were treated with high-dose chemotherapy followed by autologous bone marrow transplantation (ABMT). Eleven patients were resistant to initial therapy. Three patients had relapsed and were still responders to second or third line therapy. Three patients had relapsed but were progressing under second or third line therapy. Pre-ABMT chemotherapy included high dose cyclophosphamide in all patients (50 mg Kg-1 day-1 bolus for 4 days), most often associated with BCNU or CCNU, aracytine and 6 thioguanine. Four patients received additional TBI (10 Gy). In 9 patients complete remission (CR) was achieved, 4 failed to respond and 4 cases were not evaluable due to early death. Among CR patients, 2 died from late toxicity, 4 relapsed between the 2nd and 5th months, but 3 patients remain in CR, off therapy at 25+, 43+, and 66+ months, including 1/11 initially resistant and 2/6 who had relapsed. There were 9 treatment related deaths: 6 due to infection, 1 cardiac failure and 2 multiorgan failure. The high complete response rate in these heavily pretreated patients suggests that there may be an indication for high dose therapy earlier in resistant HD. Moreover under such conditions, treatment related morbidity would be expected to be lower.


					
Br. J. Cancer (1986), 53, 737-742

High dose chemotherapy and autologous bone marrow
transplantation in refractory Hodgkin's disease

T. Philip1, J. Dumont2, F. Teillet3, D. Maraninchi4, N.C. Gorin5, M. Kuentz6,
J.L. Harousseau7, M. Marty8, R. Pinkerton1, &                 P. Herve9

1Centre Leon Berard, 28, rue Laennec 69008 Lyon, Cedex 2; 2Institut Curie, 26, rue d'Ulm, 75231 Paris,

Cdex 05; 3Hopital Louis Mourier, 178, rue des Renouillers, 92700 Colombes; 4Institut Paoli-Calmettes, 232,
Bd Ste Marguerite, 13273 Marseille; 'H6pital St Antoine, 184, rue du Fg St Antoine, 75571 Paris, Cedex 12;

6H6pital Henri Mondor, 51, Avenue Delattre de Tassigny, 94000 Creteil; 7CHU de Nantes, Place Alexis

Ricrodeau, 44035 Nantes, Cedex; 8H6pital Saint Louis, 2 place du Dr Fournier, 75010 Paris; 9Centre Regional
de Transfusion Sanguine, I Bd A. Fleming, 25020 Besancon, Cdex.

Summary Seventeen patients with Hodgkin's disease (HD) were treated with high-dose chemotherapy
followed by autologous bone marrow transplantation (ABMT). Eleven patients were resistant to initial
therapy. Three patients had relapsed and were still responders to second or third line therapy. Three patients
had relapsed but were progressing under second or third line therapy. Pre-ABMT chemotherapy included
high dose cyclophosphamide in all patients (50 mg Kg- I day- 1 bolus for 4 days), most often associated with
BCNU or CCNU, aracytine and 6 thioguanine. Four patients received additional TBI (10 Gy). In 9 patients
complete remission (CR) was achieved, 4 failed to respond and 4 cases were not evaluable due to early death.
Among CR patients, 2 died from late toxicity, 4 relapsed between the 2nd and 5th months, but 3 patients
remain in CR, off therapy at 25+, 43+, and 66 + months, including 1/11 initially resistant and 2/6 who had
relapsed. There were 9 treatment related deaths: 6 due to infection, 1 cardiac failure and 2 multiorgan failure.

The high complete response rate in these heavily pretreated patients suggests that there may be an
indication for high dose therapy earlier in resistant HD. Moreover under such conditions, treatment related
morbidity would be expected to be lower.

Hodgkin's disease (HD) is one of the most therapy-
sensitive malignant lymphomas and in several long
term studies more than 80% of the patients have
been considered cured following radiotherapy,
chemotherapy or usually a combination of both.
Nevertheless, irrespective of age, initial presenta-
tion, stage or histologic subgroup, a few patients
remain resistant to treatment. These can present,
either with initial resistance, an incomplete response
to chemotherapy, or early and often multiple,
relapses (Teillet-Thiebaud et al., 1984). For this
small group of refractory patients, even though
some response can be obtained by changing the
therapeutic regimen, survival is very poor (Boccacio
et al., 1983). As HD is highly chemosensitive,
several teams have examined the role of high-dose
combined modality therapy for these patients, with
the support of autologous bone marrow trans-
plantation (ABMT). However, reported results have
been rather rare, due to the small number of cases,
and are usually part of larger series which include
a variety of solid tumours or haematological malig-
nancies (Jagannath et al., 1984; Phillips, 1983a,b;
Spitzer et al., 1983; Schmeizer, 1983).

Correspondence: T. Philip.

Received 5 January 1986; and in revised form, 28
February 1986.

In order to have a clearer idea of the results of
ABMT in HD, an inquiry was made among French
teams, by the French Autologous Bone Marrow
Grafting Group. Results of this study are reported
herein and include 17 cases treated in 9 different
centres.

Materials and methods
Patients

Clinical data and prior therapy are summarized in
Table I. One case has been previously reported
(Gorin et al., 1981). Ages were between 10 and 45
(median 22). Stages at diagnosis included IA (1),
IIA (2), IIB (2), IIIA (2), IIIB (1), and IVB (9).
Among the IVB patients, 2 had initial bone marrow
involvement and 8 had lung involvement.

Eleven patients were considered to have
refractory disease and had never achieved complete
remission despite combination chemotherapy and
radiotherapy (see details in Table I). The other six
had relapsed from CR (1st to the 6th relapses), 2
off therapy and 4 on therapy, and presented at time
of ABMT with disease still responsive to rescue
protocols (cases 3, 5 and 17) or not responsive to
rescue protocols (cases 4, 6 and 7). Patients had

?) The Macmillan Press, 1986

738     T. PHILIP et al.

Table I Patients treated by high dose chemotherapy and autologous bone marrow transplantation

Response to the last
Stage at        First line              Treatment at relapse   salvage therapy
Case  Age/Sex  diagnosis       treatment    Response    (salvage therapy)      prior to ABMT

1    17/M   IV B BM        MOPPx6            PR     ABVD x 6                     PD
2    36/M   IV B Lung      MOPPx6            PR     ABVDx6                       PD
3    22/F   III A          MOPPx6+RT         CR     MOPP x 6, ABVD x 3           PR
4    21/M   IV B Lung      MOPPx6+RT         CR     CVPP CCNU, ABVD x 8          PD
5    15/M   III A          MOPPx6+RT         CR     ABVDx4                       PR
6    24/M   II A           MOPPx3+RT         CR     MOPPx3, ABVDx3               PD
7    27/M   IV B Lung      MOPPx4            CR     ABVDx3,MOPPx3                PD

ABVD x 4 + RT

8    22/M   IV B Lung      MOPPx6            PR     RT                           PD
9    27/M   IV B Lung      MOPPx6            PR     CVPP x 10, Eldesine, + RT    PD
10    34/F   II A           MOPP x 9          PR     ABVD x 2, Eldesine, + RT     PD
11    12/F   IV B Lung      MOPP x 4          PR     ABVD x 4                     PD
12    16/M   IIE B          MOPP x 3          PR     ABVDx3+RT                    PD
13    31/M   IV B Lung, BM  MOPPx6            PR     ABVDx6                       PD
14    33/M   IV B Lung      MOPP x 6          PR     ABVD x 6                     PD
15    45/M   II B           MOPPx4           NR      ABVDx4+RT                    NR
16    35/M   III B          MOPPx4            PR     ABVDx2                       PD
17    13/M   I A            MOPPx4+RT         CR     ABVDx6                       CR
CR  complete remission; PR = partial response; NR =no response; RT = radiotherapy.

been treated for a median period of 24 months
prior to ABMT.

At the time of grafting, stages were 11 (3), III (2)
and IV (12) (Table I). All patients except three
(Table I) were progressing at time of ABMT.

Bone marrow freezing and storage

All patients had uninvolved bone marrow at the
time of harvesting and in vitro purging was not
attempted. The harvest was done under general
anaesthesia and in all cases bone marrow was
cryopreserved according to the usual procedure
(Gorin et al., 1983, Herve et al., 1981). The number
of nucleated cells collected for each case is
described in Table II. For some patients, 2 harvests
were necessary, due to prolonged previous therapy.

High dose therapy and autologous engraftment

Several types of combined modality therapy were
used, the details of which are summarized in
Table III. Each patient except one received
cyclophosphamide and apart from 2, all received
either CCNU or BCNU. Nine patients received the
TACC or BACT combinations, as often used in
non-Hodgkin's lymphomas, and one of these also

received TBI (12 Gy). The 8 other patients were
given various regimens, as detailed in Table III.
Three of these 8 were given TBI (1O Gy). Bone
marrow was reinfused 48h after the last dose of
cyclophosphamide, 12h after TBI and 72h after
BCNU or CCNU. Patients received a median of
1.2 x108 nucleated cells kg-1 and were nursed in
single rooms until haematologic reconstitution.

Post-treatment evaluation

Patients were evaluated at day 30 and thereafter on
a monthly basis. They were considered to have had
a complete remission only if clinical, radiology and
laboratory tests, including the sedimentation rate,
became normal. Patients who died less than 30 days
after the first day of treatment were not considered
evaluable for tumour response unless there was
clear evidence of progressive disease or autopsy
evidence of remission. No patient received main-
tenance chemotherapy after ABMT.

Results

Anti-tumour effect (Table IV)

Four patients were not evaluable (death on day 4,

HIGH DOSE CHEMOTHERAPY AND BMT IN HODGKIN'S DISEASE  739

Table II Number of nucleated cells reinfused, time to hematologic revocery, and other toxicity

<1000 WBCmm-3      <50000 p1 mm-3

Case no.       x 1O8 kg           (days)            (days)      Other toxicities

1            1.5           32                 28            Haemolytic uraemic syndrome
2            1.2            NE (>6)           NE (> 6)      Cardiomyopathy'
3            1.3            18                16            Herpes simplex

4            1              NE (>15)          NE (>15)      Cardiomyopathy; renal failurea
5            1.7            12                12            Herpes simplex

6            0.5            11                NE (> 20)     Septicemia, gastric ulcer, CNS complicationsa
7            0.9            17                22            Septicemia, lung fibrosisa
8            1.9            17                19            Vomiting

9            1              19                20            Septicemia, herpes, cardiomyopathy
10            1              18                 8            Herpes, encephalitis
11           2              NE (>21)           NE (>21)      Septicemiaa

12            1.2           39                 13            Pneumocystis

13            1.5           25                 30            Late pulmonary disease (CMV) after recovery'
14           0.5            NE (>4)            NE (>4)       Septicemiaa

15            1.5           20                 NE (>35)      (Aspergillosis)a
16           0.02           NE (>21)           NE (>21)      Renal failurea

17            1.5           NE (> 17)          NE (> 17)    (Aspergillosis)a
aFatal.

Table Ill Summary of protocols

TACC     BACT     VACC     CAC     COAC    COMAC     COCM      CCP   VPJ6-C   VPJ6-A-M
Cyclophosphamide      *        0        0       *        *        0        *       *        0
Aracytin              *        0        *

CCNU                                    0       *        *        *        0       O
BCNU

6-Thioguanine         *        a

Vindesine                               *                                  O
Vincristine                                              0        0
Adriamycin                                       *       *        a
Caryolysine                                                       a

Melphalan                                                                  0

Procarbazide                                                                       a

Etoposide                                                                                   0        0

Cyclophosphamide 40-S0mgkg-'; aracytin 200-400mgm-2; B or CCNU 200-400mgm-2; 6 thioguanine 200-
400 mgm -2; vindesine 2.5 mgm -2; vincristine 2mgm-2; adriamycin 60-100mgm-2; caryolysine 6mgm -2; melphalan
100-140mgm-2; procarbazine 200mgm-2; etoposide 100-6mgm-g2.

6, 17 and 20); 3 of these, however, showed a partial
clinical remission before death. Four patients failed
to respond and died within the first month with
progressive disease. Nine achieved CR, although 2
of these died from treatment related toxicity (cases
4 and 13), but CR was confirmed at autopsy. Four
out of the 8 survivors subsequently relapsed at 3, 4
and 5 months and died within 12 to 17 months.
Nevertheless, 3 long-term survivors are presently
free of disease, and off therapy for more than 25,
43 and 66 months, respectively.

Toxicity (Table II)

Haematopoietic reconstitution is detailed in
Table II.

Seven patients had culture proven sepsis either
during the period of aplasia (5) or within the
following 3 months (2). Organisms isolated included
2 pseudomonas, 2 escherichia coli, 2 aspergillosis,
and 1 cytomegalovirus and were lethal in 6
patients. In 1 case, an acute cardio-respiratory
failure developed possibly due to the toxicity of a

740    T. PHILIP et al.

I~~~~r~~ I-                      -E

O     >  > =  =: C:Q>>:  :   :15

X -                   _  _  O   _-o  -

0

_  ._o V  V FV U V V  V vV S U +

0.         <6 6<<!t  6   <   <   V6<  0 00  A4 0.
r~~~~~~              U HVmmV> V V V ;> >

00~~~

=  +                    ,

+                        +HE
o  W  ; o-HHQQ + +   + +   + +

+~~~~~~

- % M        > + +4)

-           .9                        a ;;;  ; ;

U cq t -> > > ;> > > >        > > >>

4-

+
aa~~~~~~~~~~~~~0

~~~~ u

0

-       - _4|)                  4))  4) I'

co    4)  Cq                     C

* 0 _  U)  )  ~  U)  Ut 0   ' 0  '0' 0 '0'0

w_ cd Cis  ct     m  Y C   c  ce-  0

C~  C,~C#~  C,~CV~UCU   CA  w  C CU)  W

O   -eU'I U Cr n   0   0  0t   0  -)  C f U r 0  0 0   0 O

0       e "0 r  , t  0   e  W r  r  E

o <  = = v =  = g g g g == = = = = v a

AGOGLUTINATION OF PERIPHERAL BLOOD LYMPHOCYTES

Histone agglutination test in microtubes. Five min reaction. x 4.

second series the test was done in microtubes
(41 x 8 mm) and evaluated macroscopically.
Both techniques gave the same results.

Test in " microtest plates '.-Lympho-
cytes were washed with Hanks' medium, and
diluted with Hanks' medium at concentra-
tions of 3000 lymphocytes/mm3. Histone
fractions (gift of Dr Johns, Chester Beatty
Institute) were dissolved in 0-145 mol/l
NaCl. Serial dilutions of histone (starting
with 10 jtg in 25 ,ul in the first well) were
then mixed with equal volumes of lympho-
cytes (i.e. 75,000 lymphocytes in 25 ul
per well). The plates were incubated for
30 min at 37?C and examined under the
microscope.

Test in microtubes.-Lymphocytes were
washed again with PBS and suspended in
PBS at a concentration of 3000 lympho-
cytes/mm3. 0-25 ml of this suspension was
distributed in microtubes (41 x 8 mm) and
20 ,tg of histone fraction was added to the
test tube. The reaction was read after
5 min and verified again after 30 min,
without incubation at 37 ?C. Where there
was a positive reaction the agglutination
of lymphocytes was clearly visible (see
Figure).

For electrophoretic mobility analysis,
the incubation at room temperature was

stopped after 5 min and cells were washed
with 0-145 mol/l NaCI, pH 7-2, and measured
in cylindrical electrophoresis apparatus as
described (Sabolovic et al., 1974).

RESULTS

Patients with various tumours were
tested, together with patients with a
variety of other diseases. They included
patients without treatment as well as
those under treatment. Moreover, some
of the patients were followed up for a 5-10
month period and retested at irregular
intervals. The controls included normal
subjects of both sexes and all ages, normal
thymus cells and tonsil lymphocytes.

Table I shows the results obtained
with all patients tested and Table II
shows the evolution of sensitivity to
agglutination in 3 individual patients.
All 5 fractions of histones (Fl, F2A1,
F2A2, F2B and F3) were tested but
positive reactions were observed only
with F2A1 and sometimes with F2A1,
F2B and F3 (Table I). 76% of all
patients were positive in our test com-
pared with negative reactions in 59

29

742    T. PHILIP et al.

F. Teillet with F. Teillet-Thiebaud, F. Courtois and F.
Meier.

H6pital Louis Mourier.

D. Maraninchi with Y. Carcassonne and J.A. Gastaut.
Institut Paoli-Calmettes.

N.C. Gorin with J.P. Laporte, A. Najman and G. Duhamel.
H6pital Saint Antoine.

M. Kuentz with F. Beaujan, J.P. Vernant and H.
Rochant.

Hopital Henri Mondor.

J.L. Harousseau with R. Garand and N. Milpied.
CHU de Nantes.

M. Marty with C. Gisselbrecht, C. Ferme and Y. Gerotat.
H6pital Saint Louis.

P. Herve with M. Flesch, J.Y. Cahn and A. Rosenbaum.
CRTS de Besancon.

References

APPELBAUM, F.R., HERZIG, G.P., ZIEGLER, J.L. & 3

others (1978). Successful engraftment of cryopreserve
autologous bone marrow in patients with malignant
lymphoma. Blood, 52, 85.

APPELBAUM, F.R., SULLIVAN, K.M., THOMAS, E.D. & 7

others (1985). Allogenic marrow transplantation in the
treatment of MOPP resistant Hodgkin's disease. J.
Clin. Oncol., 3, 1490.

BOCCACIO, C., DRONY, S., MORICEAU, M. & TEILLET, F.

(1983). Les echecs du traitement initial de la maladie
de Hodgkin. Essai de definition et approche d'un
depistage precoce. Nouvelle Revue Hematol., 25, 192.

BONADONNA, G., SANTORO, A. & BONFANTE, V. (1982).

Cyclic delivery of MOPP and ABVD combinations in
stage IV Hodgkin's disease. Rationale background
studies and recent results. Cancer Treat. Rep., 66, 881.

CANELLOS, P. (1985). Bone marrow transplantation as

salvage therapy in advanced Hodgkin disease:
allogenic or autologous. J. Clin. Oncol., 11, 1451.

CARELLA, A.M., SANTINI, G., FRASSONI, F. & 4 others

(1983).  Autologous   nonfrozen  bone   marrow
transplantation after intensive chemotherapy. A pilot
study on 18 cases. Haematologica, 68, 620.

CARELLA, A.M., SANTINI, G., GIORDANO, D. & 4 others

(1984). High-dose chemotherapy and non-frozen
autologous bone marrow transplantation in relapsed
advanced  lymphomas    or   those  resistant  to
conventional chemotherapy. Cancer, 54, 2836.

CORRINGHAM, R., GILMORE, M., PRENTICE, H.G. &

BOESEN, E. (1983). High-dose melphalan with
autologous bone marrow transplant. Cancer, 52, 1783.

DUMONT, J., PHILIP, T., MARANINCHI, D. & 6 others

(1984). High-dose chemotherapy and autologus bone
marrow transplantation in refractory Hodgkin's
disease. European Society for Medical Oncology.
Cancer Immunol. Immunother., 18, 612.

FERME, C., TEILLET, F., D'AGAY, M.F. & 3 others (1984).

Combined modality in Hodgkin's disease. Comparison
of six versus three courses of MOPP with clinical and
surgical restaging.

GOLDSTONE, A.H. (1984). Autologous bone marrow

transplantation for non-Hodgkin's lymphoma: the
preliminary European experience. In Autologous Bone
Marrow Transplantation, Proceedingv of the First
International Symposium, Dicke, K.A. and 2 others (eds)
p. 67. University of Texas, M.D. Anderson Hospital
and Tumour Institute: Houston.

GORIN, N.C., DAVID, R., STACHOWIAK, J. & 7 others

(1981). High-dose chemotherapy and autologous bone
marrow transplantation in acute leukemias, malignant
lymphomas and solid tumors. Eur. J. Cancer, 17, 557.

GORIN, N.C., NAJMAN, A., DOUAY, L. & 6 others (1983).

Lymphomes    malin   non   Hodgkiniens.  Interet
therapeutique de l'autogreffe de moelle osseuse. La
Presse Medicale, 12, 1917.

HERVE, P., ROZENBAUM, A., PLOUVIER, E. & 8 others

(1981). Greffe de cellules souches hematopoietiques
autologues. Premiers resultats d'un protocole regional.
La Presse Medicale, 10, 2001.

JAGANNATH, S., DICKE, K.A., SPITZER, G. & 4 others

(1984). Role of autologous transplantation in
Hodgkin's disease. In Autologous Bone Marrow
Transplantation, Proceedings of the First International
Symposium, Dicke, K.A. & 2 others (eds) p. 83.
University of Texas, M.D. Anderson Hospital and
Tumour Institute: Houston.

PHILIP, T., BIRON, P., HERVE, P. & 7 others (1983).

Massive BACT chemotherapy with autologous bone
marrow transplantation in 17 cases of non Hodgkin's
malignant lymphoma with a very bad prognosis. Eur.
J. Cancer Clin. Oncol., 19, 1371.

PHILIP, T., BIRON, P., MARANINCHI, D. & 6 others

(1984). Role of massive chemotherapy and ABMT in
non Hodgkin malignant lymphoma. Lancet, ii, 391.

PHILIP, T., BIRON, P., MARANINCHI, D. & 6 others

(1985). Massive chemotherapy with ABMT in 50 cases
of bad prognosis non Hodgkin's lymphoma. Br. J.
Hematol., 60, 405.

PHILLIPS, G.L. (1983a). Current clinical trial with

intensive therapy and autologous bone marrow
transplantation (ABMT) for lymphomas and solid
tumors.  Recent   Advances  in   Bone   Marrow
Transplantation, p. 567. A.R. Liss: New York.

PHILLIPS, G.L., FAY, J.W., HERZIG, G.P. & 6 others

(1983b). Intensive 1,3 Bis (2-chloroethyl) l-Nitrosourea
(BCNU), and cryopreserved autologous marrow
transplantation for refractory cancer. Cancer, 52, 1792.
PHILLIPS, G.L., HERZIG, R.H., LAZARUS, H.M. & 5 others

(1984). Treatment of resistant malignant lymphoma
with cyclophosphamide, total body irradiation, and
transplantation of cryopreserved autologous marrow.
N. Engl. J. Med., 310, 1557.

SANTORO, A., BONFANTE, V. & BONADONNA, G. (1982).

Third-line chemotherapy with CCNU, etoposide and
prednimustine (CEP) in Hodgkin's disease resistant to
MOPP and ABVD. Proc. Am. Assoc. Cancer Res., 23,
118.

SCHMEIZER, T. (1983). Autologous bone marrow

transplantation (ABMT) in solid tumors. Blut, 48, 53.

SPITZER, G., ZANDER, A., TANNIR, Z. & 4 others (1983).

Autologous bone marrow transplantation in human
solid tumors. Recent Advances in Bone Marrow
transplantation. p. 615. A.R. Liss: New York.

TEILLET-THIEBAUD, F., ASSELAIN, B., BERNARD, J. &

TEILLET, F. (1984). Failures in the treatment of
Hodgkin's disease. A survey of 36 cases out of 1014
treated  patients.  Proceedings  of  the  Second
International Conference on Malignant Lymphoma.
Lugano, Italy, p. 129 (Abstract).

				


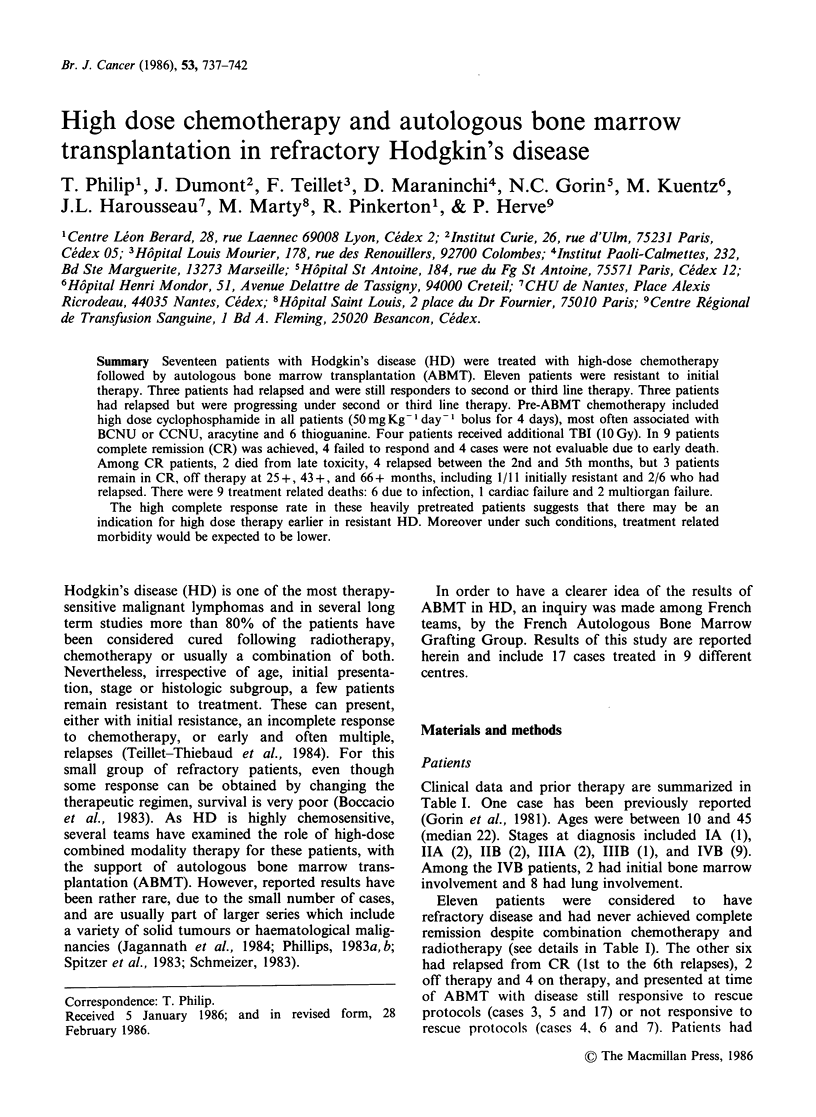

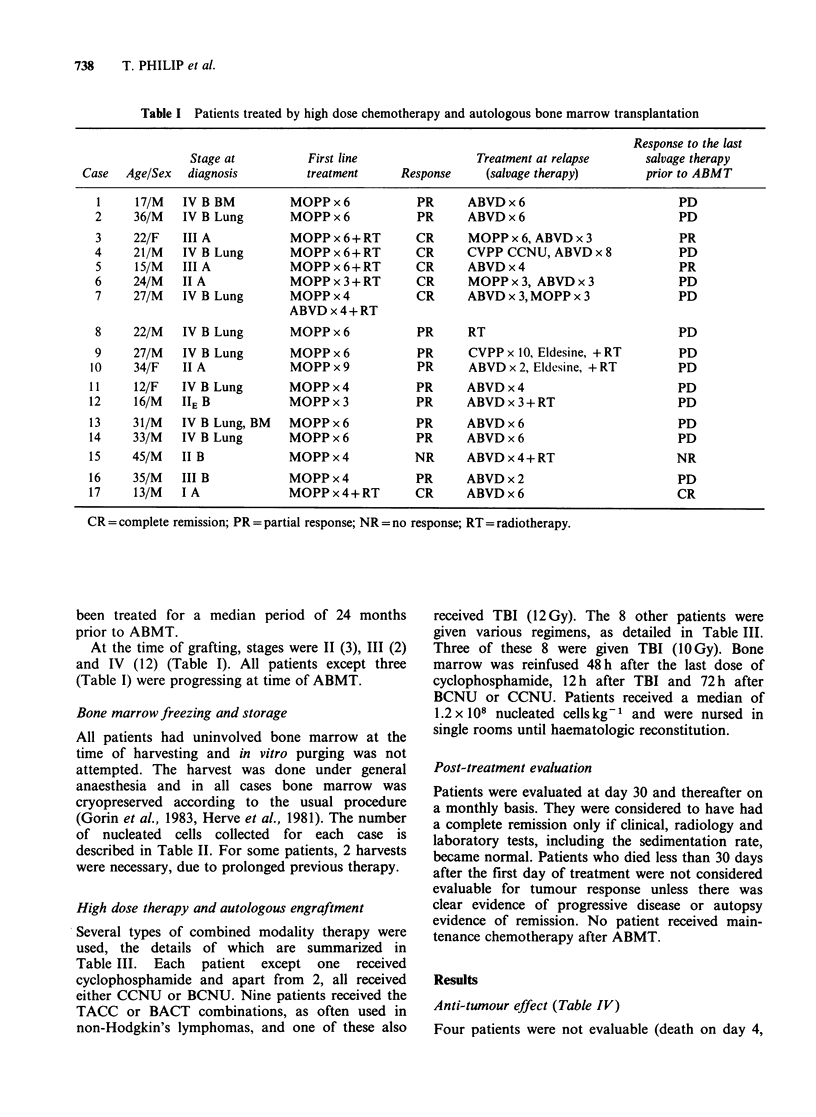

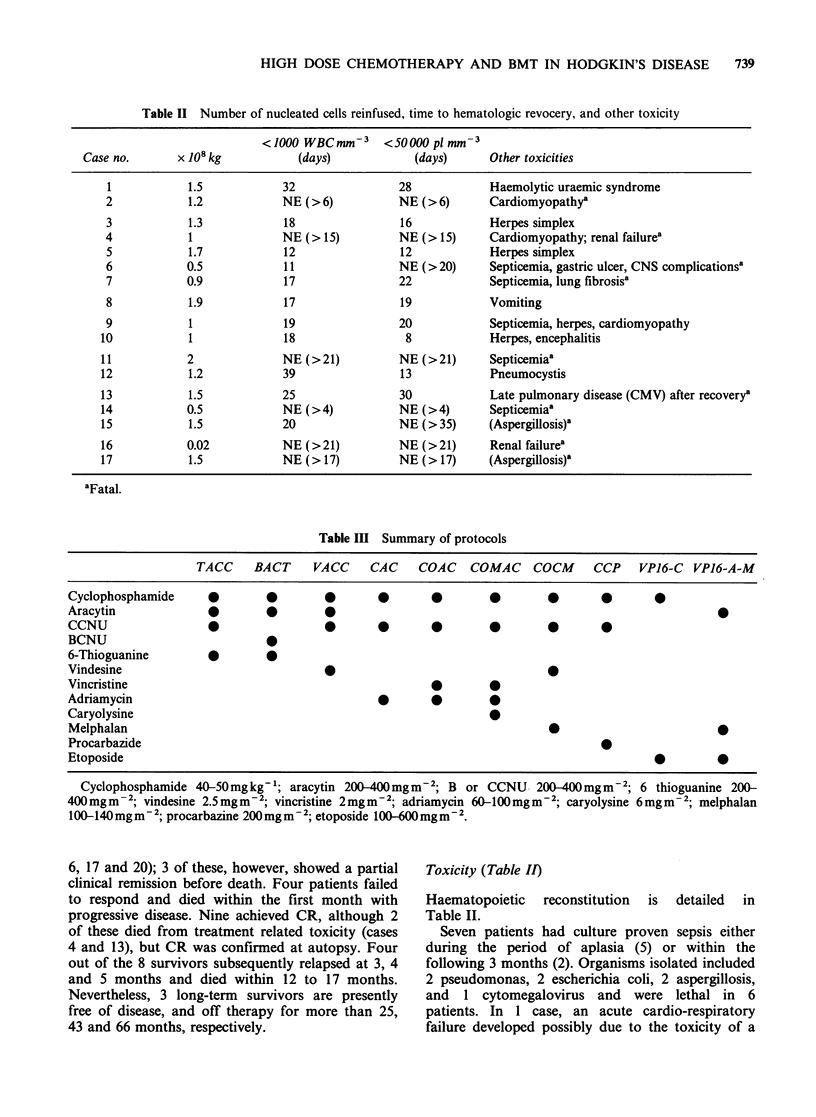

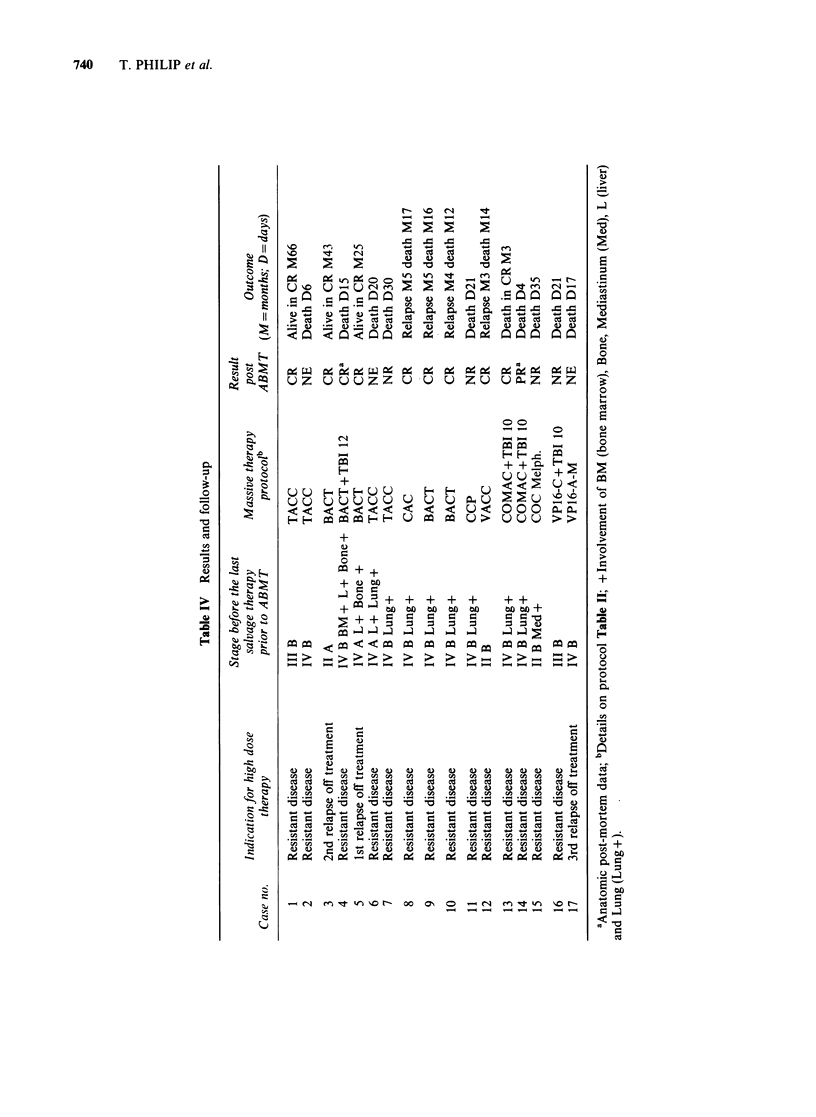

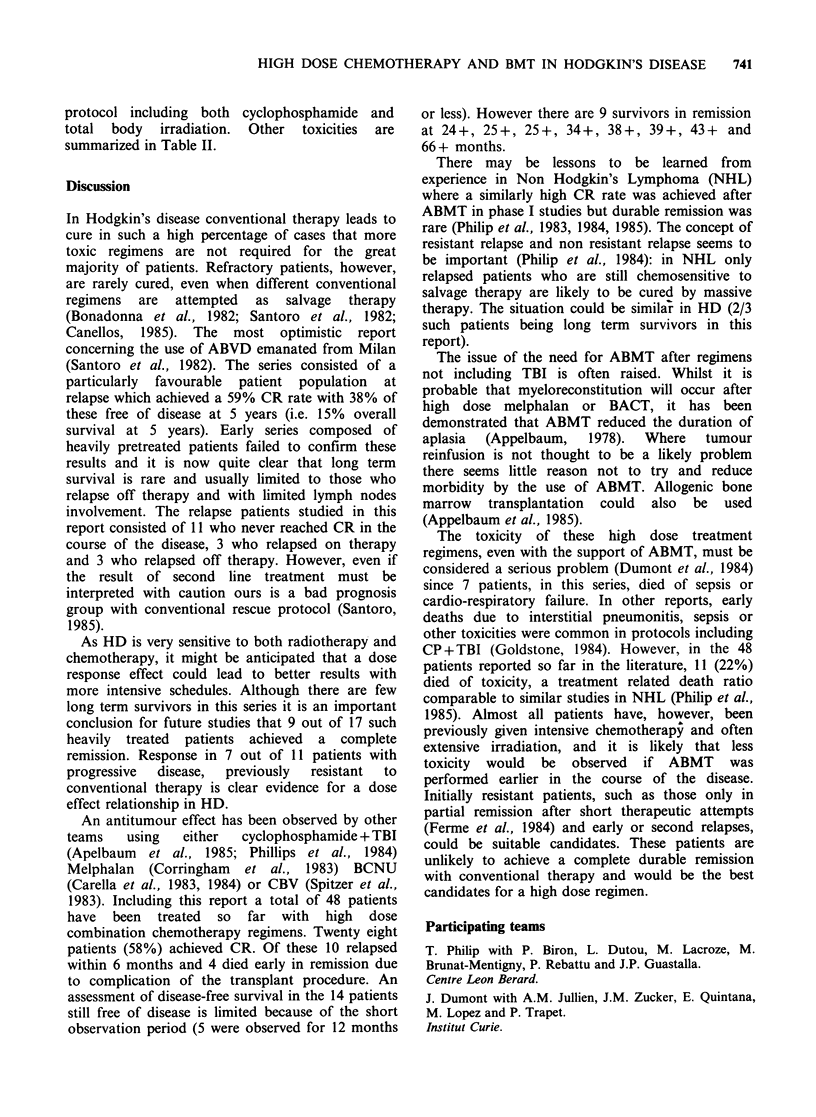

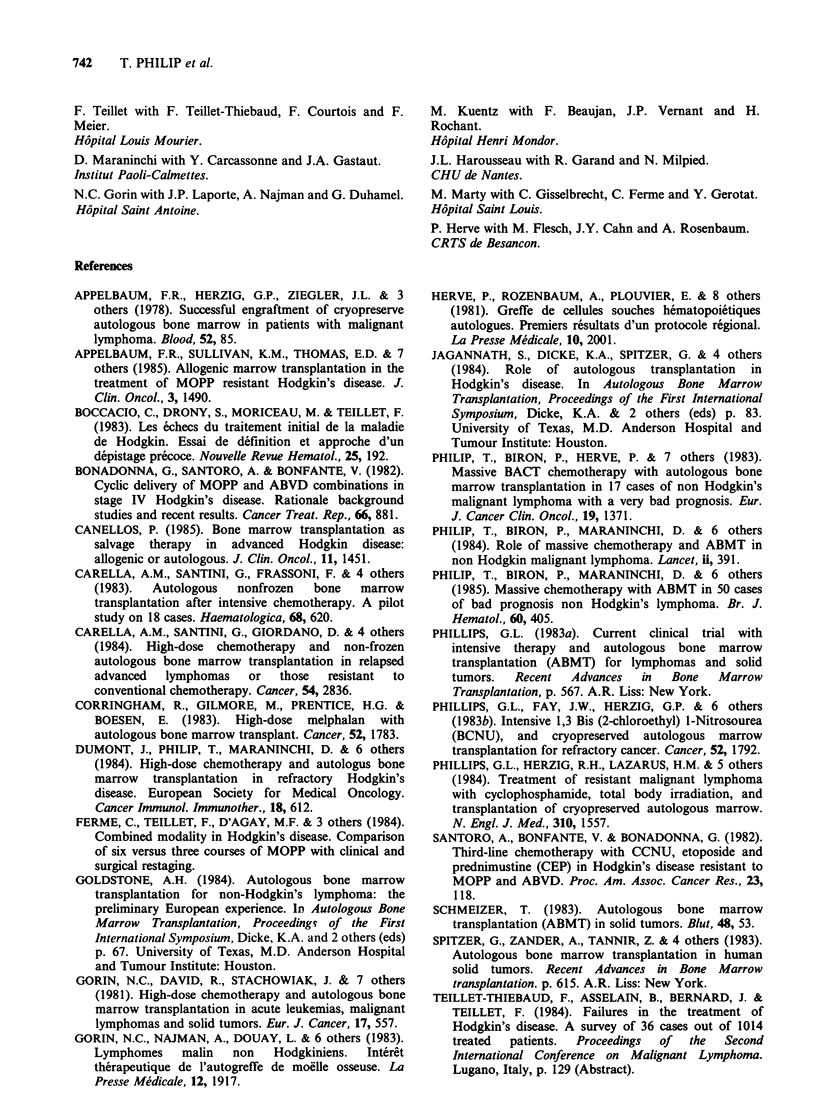

